# Mouse intestinal microbiota reduction favors local intestinal immunity triggered by antigens displayed in *Bacillus subtilis* biofilm

**DOI:** 10.1186/s12934-018-1030-8

**Published:** 2018-11-26

**Authors:** Cédric M. Vogt, Monika Hilbe, Mathias Ackermann, Claudio Aguilar, Catherine Eichwald

**Affiliations:** 1 Institute of Virology, University of Zurich, Winterthurerstrasse 266a, 8057 Zurich, Switzerland; 2 Laboratory for Animal Model Pathology, Institute of Pathology, Vetsuisse, University of Zurich, Zurich, Switzerland; 3 rqmicro AG, Schlieren, Switzerland

**Keywords:** *Bacillus subtilis*, TasA, Biofilm, Spores, Oral immunization, IgA, Antigen, Microbiota

## Abstract

**Background:**

We previously engineered *Bacillus subtilis* to express an antigen of interest fused to TasA in a biofilm. *B. subtilis* has several properties such as sporulation, biofilm formation and probiotic ability that were used for the oral application of recombinant spores harboring *Echinococcus granulosus* paramyosin and tropomyosin immunogenic peptides that resulted in the elicitation of a specific humoral immune response in a dog model.

**Results:**

In order to advance our understanding of the research in oral immunization practices using recombinant *B. subtilis* spores, we describe here an affordable animal model. In this study, we show clear evidence indicating that a niche is required for *B. subtilis* recombinant spores to colonize the densely populated mice intestinal microbiota. The reduction of intestinal microbiota with an antibiotic treatment resulted in a positive elicitation of local humoral immune response in BALB/c mice after oral application of recombinant *B. subtilis* spores harboring TasA fused to *E. granulosus* (102-207) EgTrp immunogenic peptide. Our results were supported by a lasting prevalence of spores in mice feces up to 50 days after immunization and by the presence of specific secretory IgA, isolated from feces, against *E. granulosus* tropomyosin.

**Conclusions:**

The reduction of mouse intestinal microbiota allowed the elicitation of a local humoral immune response in mice after oral application with spores of *B. subtilis* harboring immunogenic peptides against *E. granulosus*.

**Electronic supplementary material:**

The online version of this article (10.1186/s12934-018-1030-8) contains supplementary material, which is available to authorized users.

## Background

*Bacillus subtili*s is a Gram-positive bacterium that has several attractive properties with high potential in bio-applications such as vaccines [1–3] or bioremediation [4, 5]. One of these properties is the capacity to form endospores upon nutrient starvation [6, 7]. The *B. subtilis* spores have been vastly adopted as a carrier in immunization strategies because of their resistance to harsh conditions as low pH, high temperature, and noxious chemicals. In this context, methods as decoration of the spores by direct fusion with different coat proteins [8, 9] or adsorption [10–12] with the antigen of interest illustrate the versatility of the spores as an antigen carrier. Another property of *B. subtilis* is the ability to form architecturally complex communities termed biofilms, which self-produce an extracellular matrix comprised of lipids, proteins exhibiting amyloid-like properties, extracellular DNA and exopolysaccharides [13]. Interestingly, evidence suggests that *B. subtilis* can develop biofilms in the gut of living organisms [14, 15]. This is true for some non-domesticated laboratory *B. subtilis* strains as NCIB 3610 [16, 17]. We recently showed that it is possible to express heterologous proteins in a *B. subtilis* biofilm by fusion to the C-terminus of the biofilm matrix protein, TasA [18]. Optimized expression of the heterologous protein was established using a *tasA/sinR* genetic background, where SinR is a repressor of the *tapA*-*sipW*-*tasA* operon, among other genes [17, 19]. A third feature corresponds to its probiotic properties in human and livestock [20–22]. By combining these properties, we recently showed that it is possible, in a dog model, to elicit a local humoral immune response against enteric antigens such as *E. granulosus* parasite [23]. In this dog model, the recombinant spores were able to bypass the stomach barrier and then form, after germination, a biofilm in the intestine that displayed an antigen within its matrix, allowing the stimulation of gut-associated lymphoid tissue (GALT) and thereby, eliciting a local humoral immune response.

In the present study, we tested the local humoral immune response in the intestine of BALB/c mice, orally applied with recombinant spores of *B. subtilis* harboring a TasA fusion to the *E. granulosus* immunogenic peptide (102-207) EgTrp. We show that the elicited intestinal humoral immune response is favored when providing a niche to recombinant spores in the intestinal microbiota.

## Results

### Oral application of recombinant *B. subtilis* spores in mice

**Fig. 1:**
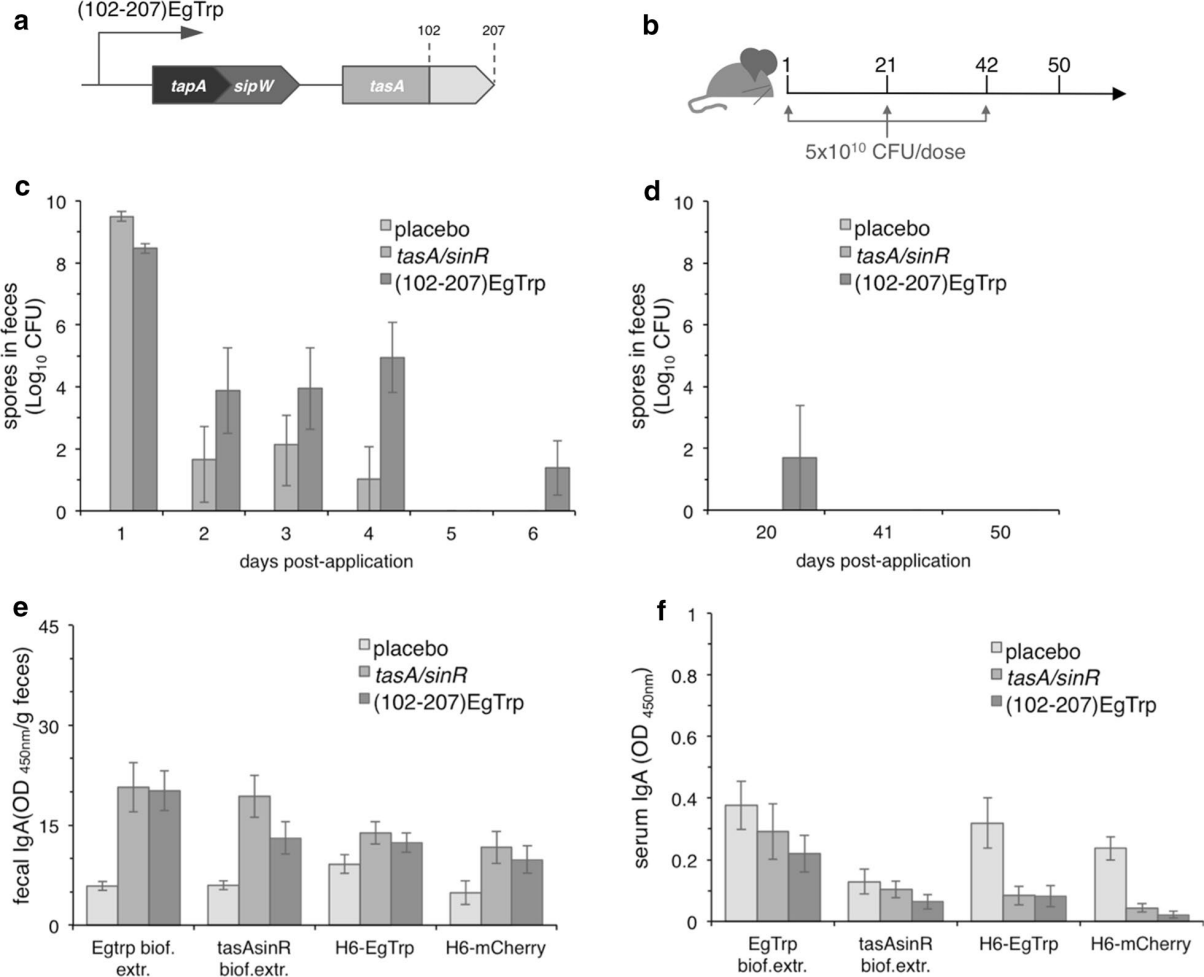
Lack of immune response after oral application with recombinant *B. subtilis* spores in mice. **a** Schematic representation of *tapA* operon carrying *E. granulosus* immunogenic peptide EgTrp fused in frame at the 3’end of *tasA*. *tapA*, anchoring and assembly protein; *sipW*, signal peptidase and *tasA*, main protein matrix. The amino acid region corresponding to the immunogenic peptide is indicated. For simplicity of the figures, TasA-(102-207)EgTrp is named as (102-207)EgTrp. Diagram not to scale. **b** Schematic schedule for the oral application of recombinant *B. subtilis* spores in mice. Three groups of six animals each were orally provided with (i) saline solution (placebo), spores of *B. subtilis tasA/sinR* and (iii) spores of *B. subtilis* (102-207)EgTrp. The animals were orally applied with 5 × 10^10^ CFU per dose on days 1, 21, 42. Feces were collected daily from days 1–6 and on days 20, 41 and 50. Quantification of a daily number of spores in feces of mice of the indicated groups after day 1 **(c)** and on days 20, 41 and 50 **(d)** post-oral application. All data are represented as mean ± SEM. The local intestinal humoral immune response, fecal sIgA **(e)** and serological IgA **(f)** were obtained by indirect ELISA coated with biofilm extract of *B. subtilis* (102-207)EgTrp, biofilm extract of *B. subtilis tasA/sinR*, recombinant H_6_-EgTrp or recombinant H_6_-mCherry. The tested animal groups are indicated

We first showed that *B. subtilis* was found in the mice intestine expressing TasA, even after the oral inoculation with recombinant *B. subtilis tasA/lux/*TasA-mCherry ([23] and Table 1) and that recombinant vegetative cells were not found in mice feces after 6 days post-inoculation (Additional file 1: Figure S1a and b). Our data also show that 64–93.5% of the orally applied recombinant *B. subtilis* spores are retained in the mice gut (Additional file 1: Figure S1c and d). Additionally, no pathologic changes, like inflammatory, degenerative or neoplastic changes were observed in the small or in the large intestine sections of all mice treated with recombinant *B. subtilis* spores when compared with untreated animals (Additional file 1: Figure S1e). Collectively, our data suggest that spores germinate in the gut of mice. To investigate if recombinant spores of *B. subtilis tasA/sinR* (102-207) EgTrp, hereafter named *B. subtilis* (102-207)EgTrp, (Fig. 1a and Table 1) can elicit a local intestinal humoral immune response in mice, we provided the recombinant spores via oral gavage. For this purpose and as depicted in Fig. 1b, three groups of mice were treated at days 1, 21 and 42 with saline solution (placebo), or with 5 × 10^10^ CFU of recombinant spores of *B. subtilis tasA/sinR* (group *tasA/sinR*) or 5 × 10^10^ CFU of recombinant spores of *B. subtilis* (102-207)EgTrp (group (102-207)EgTrp). Mice were isolated into individual cages to determine the number of shed spores after oral application and feces were collected every 24 h until day 6 and also on days 20, 41 and 50 of the scheduled treatment. As observed in Fig. 1c, mice applied with recombinant spores of *B. subtilis tasA/sinR* or (102-207)EgTrp exhibited a decline in the shedding of recombinant spores after 4 days post-application. Additionally, we estimated that the total number of recombinant spores of *B. subtilis tasA/sinR* or (102-207) EgTrp strains retained in the gut correspond to 91% and 99%, respectively. We could not detect recombinant spores in the feces of animals before the subsequent oral applications (days 21 and 42) or at experiment termination (day 50) (Fig. 1d). Next, we tested if mice could elicit a humoral intestinal immune response against *E. granulosus* antigen EgTrp by testing immunoglobulins extracted from feces at day 50 through indirect ELISA coated with *B. subtilis* biofilm extracts expressing (102-207)EgTrp antigen, biofilm extract of *B. subtilis tasA/sinR*, recombinant H_6_-EgTrp peptide or an irrelevant antigen such is H_6_-mCherry. None of the experimental animal groups showed a positive elicitation of the local humoral immune response against the tested antigens (Fig. 1e). Similar results were obtained when tested the mice sera of day 50, which unveil no elicitation of the humoral immune response of neither IgG (Additional file 2: Figure S2a) nor IgA (Fig. 1f). Also, the animals remained healthy during the whole procedure as denoted by the lack of difference in the mean body weight among the experimental groups (Additional file 2: Figure S2c) and the absence of symptoms such as diarrhea, hypothermia, and mastocytosis. Thus, the recombinant spores of *B. subtilis* seem not able to elicit an immune response under the conditions tested in these experiments.

**Table 1:** *Bacillus subtilis* strains used in this study

Strain	Genotype^a^	Reference/ source
*tasA/SinR*	*tasA*-*sinR*::Km^r^	Vogt et al. [18]
*tasA/sinR*/TasA-(102-207)EgTrp	*tasA*-*sinR*::Km^r^; *amyE*::*yqxM*-*sipM*-*tasA*-(102-207)EgTrp Spc^r^	Vogt et al. [18]
wt/*lux*	*lacA::*P_*tasA*_-*luxCDABE* Erm^r^	Vogt et al. [23]
*tasA/lux/*TasA-mCherry	*tasA*::Km^r^*; lacA::*P_*tasA*_-*luxCDABE* MLS^r^*; AmyE::yqxM*-*sipW*-*tasA*-mCherry Cm^r^	Vogt et al. [23]
*tasA/sinR/lux/*TasA-mCherry	*tasA*::Km^r^*SinR::*Spc^r^*; lacA*::P_*tasA*_-*luxCDABE* MLS^r^*; AmyE::yqxM*-*sipW*-*tasA*-mCherry Cm^r^	Vogt et al. [23]

^a^ Km^r^: kanamycin resistance; Spc^r^: spectinomycin resistance; Cm^r^: chloramphenicol resistance; MLS^r^: macrolide-lincosamide-streptogramin B (erythromycin and lincomycin) resistance

### Reduction of the intestinal microbiota favors local immune response after oral application of recombinant *B. subtilis* spores in mice

**Fig. 2:**
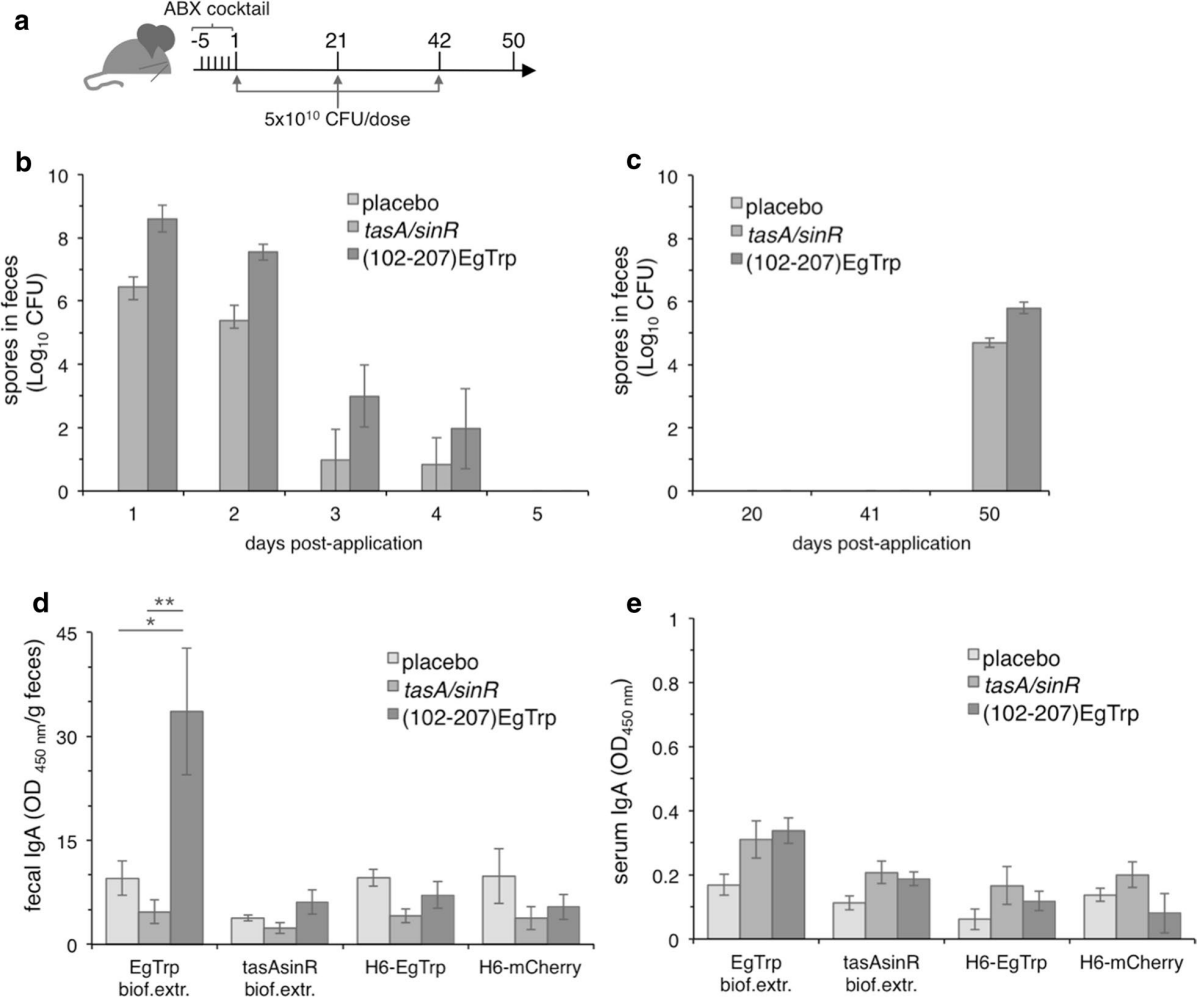
Reduction of intestinal microbiota elicits a local humoral immune response. **a** Schematic schedule for the oral application of recombinant *B. subtilis* spores in mice. All the animals were treated with an antibiotic (ABX) cocktail for 5 days before the first application of recombinant *B. subtilis* spores. Three groups of six BALB/c mice each were orally provided with (i) saline solution (placebo), (ii) spores of *B. subtilis tasA/sinR* and (iii) spores of *B. subtilis* (102-207)EgTrp. The animals were orally applied with 5 × 10^10^ CFU per dose on days 1, 21, 42. Feces were collected daily from days 1–6 and on days 20, 41 and 50. Blood samples were collected on days 1, 21, 42 and 50. Quantification of the daily total number of spores (CFU) in feces of mice of the indicated groups after **(b)** day 1 and on **(c)** days 20, 41 and 50 post-oral application. The data represent the mean ± SEM. Determination of the local intestinal humoral immune response, fecal sIgA **(d)** and serological IgA **(e)** using indirect ELISA coated with biofilm extract of *B. subtilis* (102-207)EgTrp, biofilm extract of *B. subtilis tasA/sinR*, recombinant H_6_-EgTrp or recombinant H_6_-mCherry. The tested animal groups are indicated. The data represent the mean ± SEM, and t-test unpaired two-tailed; * p < 0.05; ** p < 0.01

To stimulate the GALT with a consequent elicitation of a humoral immune response against the presented antigens, the spores of *B. subtilis* require a niche where to germinate, colonize and form a biofilm. However, the efficiency of this process may be severely hampered by the densely populated host intestinal microbiota, which could be actively competing for the same niche. We hypothesized that this competition could be of different nature such as for nutrient availability [24] or space competition, impeding the colonization of new bacterial species [25]. In any of these circumstances, the reduction of the intestinal microbiota before the oral application of recombinant spores of *B. subtilis* could provide a chance for successful colonization and subsequent biofilm formation, which in turn may elicit a local humoral immune response. To test this hypothesis, we treated mice for five consecutive days with an antibiotic cocktail [26]. The effective reduction of the intestinal microbiota was corroborated by the absence of bacterial colonies isolated from feces in three different culture media such as Luria-Bertani, brain heart infusion and nutrient broth (Additional file 3: Figure S3 a, b and c). Of note, the antibiotic treatment had no detrimental effect on the mice health as denoted by similar bodyweight among untreated and treated animals (Additional file 3: Figure S3d). Thus, as described in Fig. 2a, all groups of mice were first treated for 5 days with an antibiotic cocktail, followed by three oral applications of 5 × 10^10^ CFU of recombinant spores per dose on days 1, 21 and 42. All animals were sacrificed on day 50. The animals were divided, as above, into three experimental groups: (i) placebo, (ii) *tasA/sinR* and (iii) (102-207)EgTrp. As in the previous experiment, mice shed recombinant spores until the fourth day from the first oral application (Fig. 2b). The number of recombinant spores retained in the gut was 99.9% for *B. subtilis tasA/sinR* and 97.6% for (102-207)EgTrp. Surprisingly, recombinant spores from both *tasA/sinR* and (102-207)EgTrp groups were readily detected at day 50 in the mice feces (Fig. 2c). The local intestinal humoral immunity was determined using an indirect ELISA coated with either an EgTrp biofilm extract, *tasA/sinR* biofilm extract, recombinant H_6_-EgTrp or recombinant H_6_-mCherry. Interestingly, the secretory IgA (sIgA) isolated from feces of mice of the (102-207)EgTrp group recognized specifically the biofilm extracts harboring the heterologous expression of the immunogenic peptide EgTrp (Fig. 2d). In the same conditions, no recognition by sIgA isolated from mice feces of the placebo and *tasA/sinR* groups was observed for EgTrp expressed in biofilm extracts. Importantly, the lack of TasA and SinR in biofilm extracts (i.e., *tasA/sinR* biofilm extracts), resulted in no local humoral response for the three tested groups. Additionally, no response for sIgA was obtained for H_6_-EgTrp or H_6_-mCherry. Mice did not induce any serological humoral immune response for the tested antigens in an IgG (Additional file 1: Figure S1b) or IgA context (Fig. 2e). Between the experimental groups, no differences were observed in animal body weight (Additional file 2: Figure S2d), as well as no signs of diarrhea, hypothermia or mastocytosis after pretreatment with antibiotics, followed by oral application of recombinant *B. subtilis* spores.

## Discussion

It is of high interest to perform antigen tests aimed at vaccine development in affordable animal models such as rodents, being mice (as BALB/c strain) frequently the model of choice. Initial evidence suggested that recombinant *B. subtilis* spores germinate in the gut of BALB/ c mice without generating adverse effects. Therefore in an attempt to evaluate the effectiveness of our method, based on the display of antigens in biofilms, we used a previously tested recombinant *B. subtilis* (102-207)EgTrp that elicited a positive humoral immune response in dogs [23]. The antigen (102-207) EgTrp corresponds to tropomyosin, an immunogenic peptide of *Echinococcus granulosus*, described previously by Pétavy and collaborators [27, 28]. *E. granulosus* cestode is the causative agent for cystic echinococcosis, responsible for high human morbidity and mortality and having a high economic impact on livestock [29–31]. In our first trial in mice, we could not detect an elicitation of the local immune response after three oral doses of recombinant *B. subtilis* spores. This observation was not surprising, and it is consistent with previous observations [9, 32–39], where even nine consecutive oral doses with *B. subtilis* spores evoked weak or null immune response compared to similar conditions after intranasal immunization in mice [8]. The pre-treatment with antibiotics to reduce the density of gut microbiota is a standard procedure in other mouse models of enteric infection [9, 40]. In our experimental setting, we administrated an antibiotic cocktail [26] for 5 days before the first oral application of recombinant *B. subtilis* spores. The persistence of the recombinant spores in the gut was determined by their prevalence in feces at day 50 post-application compared with the mice untreated with antibiotics in the same conditions. The spore persistence suggests that recombinant *B. subtilis* spores were able to find a niche in the gut (colonization), followed by germination, biofilm formation, and subsequent sporulation. Additionally, an elicitation of local humoral immune response as determined by the presence of specific sIgA recognizing EgTrp. Therefore, the decrease of the intestinal microbiota of mice, colonized for at least 1000 different bacterial species (40), is a requisite for the settlement of recombinant *B. subtilis* spores. Previous evidence demonstrates that *B. subtilis* naturally colonize the gut of humans [14], dogs [23], and even grass carps [41], suggesting that *B. subtilis* cannot be considered as an allochthonous microorganism. Interestingly, the generated immunoglobulins recognized the EgTrp antigen peptides only when present in a biofilm extract but not the one from the recombinant purified H_6_-(102-278)EgTrp. In this context, we recently showed [23] that dogs treated orally with recombinant spores of *B. subtilis* (102-207)Egtrp elicited a humoral immune response recognizing specifically recombinant purified H_6_-EgTrp. Our results suggest that the biofilm TasA-(102-207)EgTrp fusion displayed in the gut mice got selected by immunoglobulins through specific conformation that is not forged by H_6_-EgTrp [42, 43]. This outcome also confirms the dissimilarities between the intestinal immune system of mice and that of dogs. It is important to highlight that the results obtained here cannot necessarily apply to a different animal model. The specificity of the humoral response was denoted by the lack of recognition of unrelated antigens as H_6_-mCherry by sIgA. It is of note that we could not distinguish a serum-specific IgA in any of the experimental settings tested. As expected, the use of recombinant *B. subtilis* spores resulted safe in mice. Our recombinant spores under the conditions described do not alter the health status of treated mice when compared to its control pairs, as denoted by no discrepancy in their body weight, pathological states (like hypothermia, diarrhea or mastocytosis) and by infiltration of inflammatory cells in the small and large intestine.

## Conclusions

In this work, we provide evidence that a reduction in the intestinal microbiota in mice could settle the basis for favoring an oral immunization using recombinant *B. subtilis* spores.

## Methods

### Ethics statement

All the mouse experiments were performed according to the guidelines of the animal experimentation law (SR 455.163; TVV) of the Swiss federal government. The Cantonal Veterinary Office of Zurich, Switzerland, approved the protocols under animal experimentation number 104/2012.

### *Bacillus subtilis* strains, media and culture conditions

The *B. subtilis strains* used in this study were previously described by Vogt et al. [18]. For routine growth and spore quantification, cells were propagated on Luria-Bertani (LB) medium. The final concentrations of antibiotics used for the *B. subtilis* strains were as follows: 100 μg/ml for spectinomycin (Spc) and 10 μg/ml for kanamycin (Km).

### Plasmid constructions

pQE32-(102-278)EgTrp and pQE32-mCherry were previously described by Vogt et al. [23].

### Production of recombinant *B. subtilis* spores

The recombinant spores were produced and purified as described by Vogt et al. [18].

### Experimental administration of recombinant *B. subtilis* spores in mice

Intragastric gavage was orally applied to groups of six mice (females, BALB/c, 6 weeks old) with 5 × 10^10^ CFU of recombinant spores of *B. subtilis* in a final volume of 200 µl PBS per dose on days 1, 21 and 42. The placebo group was orally applied with 200 µl PBS. Mice were isolated into single cages for collection of feces every 24 h, on days 1 to 6 and on days 20, 41 and 50. Blood samples were collected by tail-bleeding the day before the application of a spores dose and at day 50. The serum was used to test the humoral response against the antigen of interest. All animals were sacrificed on day 50 post-application.

### Mouse intestinal microbiota reduction

When indicated, mice were treated with an antibiotic mixture for the elimination of the gut microbiota as described by Shan et al. [26]. For this purpose, a mixture containing 0.5 mg/ml ampicillin (PanReac AppliChem, Spain), 0.5 mg/ml gentamycin (PanReac AppliChem, Spain), 0.25 mg/ml vancomycin (Alfa Aesar, Germany), metronidazole (Alfa Aesar, Germany), and 20 mg/ml sucrose (Sigma) dissolved in sterile drinking water was provided as source of water to mice for 5 days. The reduction of the intestinal microbiota was monitored by counting the number of bacterial colonies isolated from feces in non-selective media, such as LB, heart-brain infusion and nutrient broth semi-solid media.

### Quantification of spores in feces

Feces of single-caged mice were collected throughout 24 h. The feces were resuspended to a concentration of 0.2 g/ml in PBS, homogenized with vortex for 30 s. Then, a 300 µl aliquot of feces resuspension was heated for 20 min at 80 °C to kill vegetative cells. Serial dilutions were plated on selective semi-solid LB agar containing 10 µg/ml kanamycin to determine viable recombinant spores. The spore number per gram of feces was obtained dividing the colony forming units (CFU) by the amount in grams of feces.

### Indirect ELISA

The assays were performed as described in detail by Vogt et al. [23]. For the extraction of secretory antibodies (sIgA), fecal samples were collected throughout 24 h from mice isolated in single cages and stored at − 20 °C until analysis. One gram of feces was resuspended in 5 ml PBS, vortex homogenized for 30 s and centrifuged at 800×*g* for 10 min. Then, 360 µl of recovered supernatant was mixed with 40 µl of feces buffer (1% BSA, 0.01% Triton X-100, 0.1% 2-mercaptoethanol and protease inhibitor (cOmplete™ EDTA-free protease inhibitor cocktail, Roche, Switzerland)) and kept in ice. Recombinant purified protein (500 ng/well) or 72 h biofilm extract (OD_600nm_ of 0.0002/well) in 0.2 M bicarbonate buffer pH 9.4 was coated for 16 h at 4 °C in a 96 well multi-well plates (Nunc-Immuno Maxisorp, Thermo Scientific). Plates were incubated for 2 h at room temperature with blocking buffer (1% BSA in PBS). A 100 µl aliquot of feces mixture was incubated for 2 h at 37 °C. This was followed by incubation of the plates for 1 h at 37 °C with goat anti-mouse IgA conjugated to peroxidase (diluted 1:600, Sigma) or rabbit anti-mouse IgG (whole molecule) conjugated to peroxidase (diluted 1:2000, Sigma) both diluted in 1% BSA-PBS. The plates were incubated with 100 µl per well of 3,3’,5,5’-tetramethylbenzidine (TMB) substrate (ThermoFisher Scientific) in the dark for 30 min at room temperature, and the reaction was stopped by the addition of 100 µl 1 M H_2_SO_4_. The plates were read using an SLT 340 ATTC Tecan microplate reader (Tecan US Inc.) at an OD_450nm_. The data were analyzed and processed using Microsoft^®^Excel^®^ for MAC 2011. The cut-off was determined as the average of three negative controls. The negative control value was obtained by incubation of the antigen followed by the secondary antibody conjugated to HRP. The cut-off was subtracted from all the sample values. Each value has been subtracted from its corresponding pre-immune (PI) value.

### Expression and purification of H_6_-tagged proteins

H_6_-EgTrp and H_6_-mCherry were expressed in *Escherichia coli* M15 (pREP4) (Qiagen) transformed with pQE32-(102-278)EgTrp and pQE32-mCherry, as described in detail by Vogt et al. [23].

### Preparation of biofilm extract

For the preparation of biofilms, cells were scraped from overnight growth on LB-agar plates, resuspended in LB liquid medium to an OD_600 nm_ of 1, and then 2 µl of this suspension was spotted on MSgg solid medium [16]. Biofilms were incubated at 30 °C. At 72 h, the biofilm was harvested in 0.2 M bicarbonate buffer pH 9.4 and dispersed using mild sonication conditions (1 min at 14 kHz) to obtain a homogeneous resuspension. For the coating of ELISA plates, biofilm extracts were normalized to OD_600nm_ of 2 × 10^−4^.

### Histology and immunohistochemistry

The mice’s intestines were sectioned in duodenum, jejunum, ileum, cecum, and colon. Each intestinal section was knotted in both ends previous to sectioning to avoid the loss of the intestinal content. Samples of approximately 2 cm in length were fixed in 4% formaldehyde. After fixation, each sample was dehydrated in alcohol solutions of increasing concentration and embedded in paraffin. The embedded samples were thin-sectioned at 2–3 µm and stained with hematoxylin and eosin.

For immunohistochemistry of the mice’s intestinal section, the samples were de-paraffinized, rehydrated and incubated for 30 min at room temperature with the primary antibody (rabbit anti-TasA serum). A detection kit, containing the secondary antibody and aminoethyl carbazole as chromogen, was subsequently applied according to the manufacturer’s protocols (Peroxidase/AEC Rabbit/Mouse Kit, DAKO). Images were acquired using an Olympus CX41 light microscope equipped with a 40X objective lens and an Olympus Vanox-S AxioCam interface. The acquired images were processed using Image J software (Wayne Rasband, NIH, USA; http://imagej.nih.gov/ij).

## Additional files

**Additional file 1: Figure S1.** Tracking of Bacillus subtilis recombinant in the gut of mice. Two mice were orally applied with 5 × 10^10^ CFU spores/dose of *B. subtilis* wild-type (wt) or tasA lux TasA-mCherry strains. (a) Feces were monitored for luminescence at 7 days post-application of recombinant spores. A color luminescence scale is shown at the right of the panel. The luminescent images were acquired with a Xenogen IVIS camera and analyzed using Living Image^®^ 4.0 software (Caliper Life Sciences, USA) (b) Immunohistochemistry of mice cecum sections incubated with anti-TasA sera (1:100) followed with secondary anti-rabbit-HRP and stained with diaminobenzidine (brown). The cell membranes and nuclei were counterstained with eosin (red)/hematoxylin (blue). Scale bar is 100 µm. (c) Schematic representation of mice oral application of recombinant *B. subtilis* spores schedule. Mice (six females BALB/c, 6 weeks old per group) were treated 5 × 10^10^ CFU spores/ dose on days 1, 21 and 42. The experimental groups were the following with (i) placebo (saline solution), (ii) wild-type, (iii) *tasA lux* TasA-mCherry and (iv) *tasA sinR lux* TasA-mCherry. On day 50, animals were sacrificed. (d) Plot of the total number of shed *B. subtilis* spores (CFU) in feces after each oral application. Data represent the mean ± SD for each group of animals. (e) Representative histological sections stained with hematoxylin and eosin from intestinal samples of dogs orally inoculated with recombinant *B. subtilis* spores (placebo, wt, *tasA* lux TasA-mCherry and *tasAsinR lux* TasA-mCherry). Lm, intestinal lumen. Scale bar is 100 µm.

**Additional file 2: Figure S2.** IgG humoral response. Determination of the serological IgG response by indirect ELISA of mice untreated (a) or pretreated with ABXs (b) before oral application of recombinant B. subtilis spores. The plates were coated with biofilm extract of B. subtilis strain (102-207)EgTrp, biofilm extract of B. subtilis tasA/sinR, recombinant purified H_6_-EgTrp or recombinant purified H_6_-mCherry. The tested animal groups are indicated. The body weight curve of the indicated mice groups untreated (c) or pretreated (d) with antibiotics before oral application with recombinant B. subtilis spores. The data represent the mean ± SEM and t-test unpaired two-tailed.

**Additional file 3: Figure S3.** Antibiotic (ABX) cocktail treatment severely reduces bacterial microflora. One mouse (untreated, solid dark circle) and two mice (ABX 1, solid dark square and ABX 2, open square) were untreated or treated with an antibiotic cocktail (ampicillin, gentamycin, vancomycin, and metronidazole) in the drinking water, respectively. Feces samples of individual cages were collected every day, diluted in PBS and cultured by serial dilution in enriched media as (a) Luria-Bertani, (b) Brain-Heart Infusion and (c) Nutrient Broth. The plots represent the number of Log10 CFU/ml from resuspended feces. (d) Plot of the body weight (g) of the animals during the 6 days of antibiotic treatment.

## Abbreviations

CFU: colony forming unit
TMB: 3,3′,5,5′-tetramethylbenzidine
LB: Luria-Bertani
sIgA: secretory immunoglobulin A
ABX: antibiotic
